# Supplementary figures and images for: Primary health care utilization in the first year after arrival by refugee sponsorship model in Ontario, Canada: A population-based cohort study
Source: PLoS One. 2023 Jul 26;18(7):e0287437. doi: 10.1371/journal.pone.0287437 (PMC10370760; doi:10.1371/journal.pone.0287437)

# S1 Fig: Database linkage and cohort selection flowchart

#
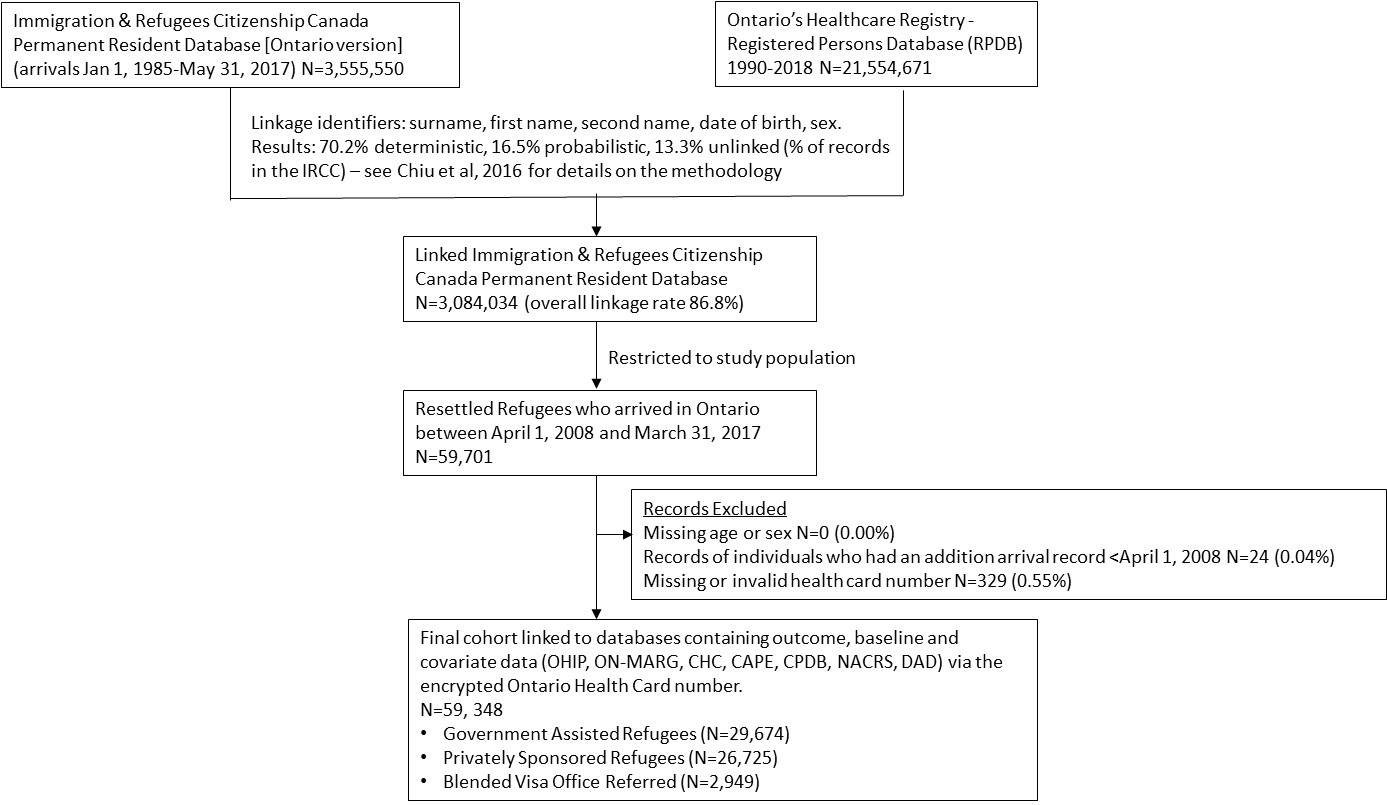

Supplement: S1 Fig — (DOCX) [file pone.0287437.s001.docx]
