# Supplementary material for: Primary health care utilization in the first year after arrival by refugee sponsorship model in Ontario, Canada: A population-based cohort study
Source: PLoS One. 2023 Jul 26;18(7):e0287437. doi: 10.1371/journal.pone.0287437 (PMC10370760; doi:10.1371/journal.pone.0287437)
Supplement: S1 Table — (DOCX) [file pone.0287437.s002.docx]

# S1 Table: Study data sources

| Data Sources | Definitions |
| --- | --- |
| Registered Persons Database (RPDB) (healthcare registry) | The RPDB provides basic demographic information about anyone who has ever received an Ontario health card number since 1990, including date of birth, age and sex. |
| Immigration, Refugees and Citizenship Canada | The Immigration, Refugees and Citizenship Canada (IRCC)’s Permanent Resident Database includes immigration application records for people who applied to land in Ontario between January 1, 1985 and May 31, 2017. The data contains permanent residents’ demographic information such as country of citizenship, level of education, mother tongue, and landing date. |
| Ontario Health Insurance Plan (OHIP) | The OHIP claims database contains information about services paid for by the Ontario Health Insurance Plan (OHIP). Services may include physician consultations and assessments in the outpatient, acute care, and long-term care settings; information on diagnostic and therapeutic procedures; surgical procedures; and laboratory services. These data only capture information for those physicians who work on a fee-for-service basis. |
| Ontario Marginalization Index (ON-MARG) | The Ontario Marginalization Index (ON-MARG) is a geographically (Census) based index developed to quantify the degree of marginalization occurring across the province of Ontario. It is comprised of 4 major dimensions thought to underlie the construct of marginalization: *residential instability, material deprivation, dependency and ethnic concentration*. |
| Community Health Centres (CHC) | Community health centres (CHCs) data includes information on primary care services provided by health care professionals notably physicians, nurse practitioners, nurses, counsellors, community workers, and dietitians. CHCs can be accessed by all residents regardless of provincial health insurance coverage or citizenship status. CHCs are primarily located in areas of high need for underserved populations. |
| ​Client Agency Program Enrolment (CAPE) | The Client Agency Program Enrolment dataset captures data on patient enrolment with physicians and groups, including, enhanced fee-for-service and capitation models. |
| Corporate Provider Database (CPDB) | The Corporate Provider Database contains information on providers/groups eligible to receive payment from OHIP. Contains information on providers at the individual level including physicians, pharmacists, optometrists, nurses etc. Also contains information on the groups to which providers belong, including primary care offices, hospitals, labs etc. |
| National Ambulatory Care Reporting System (NACRS) | The National Ambulatory Care Reporting System (NACRS) captures data on patient visits to hospital and community based ambulatory care, including day surgery, outpatient clinics and emergency departments. |
| Discharge Abstract Database (DAD) | The Discharge Abstract Database (DAD) includes patient-level information for acute, rehab, chronic, and day surgery institutions in Ontario. The DAD captures information on patient separations such as clinical (diagnoses, procedures) and administrative data (admission category, length of stay, and discharge disposition). |
| Same Day Surgery (SDS) | The Same Day Surgery (SDS) database contains chart abstraction information for all same-day surgeries performed at acute care hospitals in Ontario. Every record corresponds to one same-day surgery or procedure stay. |
