# Supplementary material for: Primary health care utilization in the first year after arrival by refugee sponsorship model in Ontario, Canada: A population-based cohort study
Source: PLoS One. 2023 Jul 26;18(7):e0287437. doi: 10.1371/journal.pone.0287437 (PMC10370760; doi:10.1371/journal.pone.0287437)
Supplement: S2 Table — (DOCX) [file pone.0287437.s003.docx]

# S2 Table: List of study variables

| **Variables Used in this study** | **Database Source** | **Variable Definition & Categories** | **Collected/**  **measured at** |
| --- | --- | --- | --- |
| **PRIMARY EXPOSURES** | | | |
| Resettled refugee category | Immigration, Refugees, and Citizenship Canada’s (IRCC) Permanent Resident Database | - Government sponsored refugees - Privately sponsored refugees - Blended visa office referred refugees | Time of immigration application |
| Era of Landing + Country of Birth | Immigration, Refugees, and Citizenship Canada’s (IRCC) Permanent Resident Database | - Pre-Syrian Era: April 1, 2008 to October 31, 2015 - Syrian Era: November 1, 2015 to March 31, 207 - Syrians – born in Syria - Non-Syrians – born in countries other than Syria | Time of immigration application |
| **SECONDARY EXPOSURE** | | | |
| Country of birth cohorts (with explicit refugee resettlement commitments or large population sizes) | Immigration, Refugees, and Citizenship Canada’s (IRCC) Permanent Resident Database | - Syria (commitment) - Bhutan (commitment) - Myanmar (commitment) - Iraq (commitment) - Afghanistan - Iran - Somalia - Eritrea - Democratic Republic of Congo - Ethiopia - All other African countries (not specified above) - All other countries (not specified above) | Time of immigration application |
| **PRIMARY OUTCOMES** | | | |
| Time to first primary care visit in the first year after landing | Ontario Health Insurance Plan (OHIP) | Time in days to first primary care visit to a family physician/general practitioner (GP), pediatrician, or nurse practitioner using previously validated physician billing fee and diagnosis codes. | First primary care visit |
| Time to any CHC visit in the first year after landing | Community Health Centres (CHC) | Time in days to any visit to a CHC captured in the electronic medical record. | First CHC visit |
| **SECONDARY OUTCOMES** | | | |
| Proportion with a primary care visit in the first two months of arrival | Ontario Health Insurance Plan (OHIP) | Primary care visit to a family physician/general practitioner (GP), pediatrician, or nurse practitioner in first two months after arrival. Uses previously validated physician billing fee and diagnosis codes and CHC medical records. | First two months after arrival |
| Proportion with a primary care visit by the end of the first year after arrival | Ontario Health Insurance Plan (OHIP) | Primary care visit to a family physician/general practitioner (GP), pediatrician, or nurse practitioner in first year after arrival. Uses previously validated physician billing fee and diagnosis codes and CHC medical records. | First year after arrival |
| Primary Care Affiliation | Client Agency Program Enrolment (CAPE)  Corporate Provider Database (CPDB)  Community Health Centres (CHC)  Ontario Health Insurance Plan (OHIP) | We assigned individuals to their primary care enrollment model or provider at the end of the first year using previously validated methods [32]  Primary Care Enrollment model assigned and categorized in the following sequence:   1. CHC – any visit to a CHC with subcategories:   a) specializing in immigrants and refugees,  b) located in a high immigrant/refugee area (>25% of the patient population),  c) other CHCs [33].   1. Comprehensive – based on the rostering list for those in a primary care enrollment model includes patients enrolled in a Family Health Group (FHG), Family Health Network (FHN), Family Health Organization (FHO) [20]. 2. Other -those without a CHC visit or not enrolled in a primary care model were assigned to the primary physician with the highest dollar value of all billings for primary care visits. This includes pediatricians and general practitioners and family physicians practicing outside of primary care models (not providing comprehensive services) and work in walk-in clinics or solo-practices 3. Pediatrician 4. Other primary care 5. No primary care – those with no primary care affiliation and no primary care claims in the 1^st^ year after arrival | At the end of the first year after arrival |
| Any major morbidity | OHIP  NACRS  DAD  SDS | Uses diagnostic codes captured in health service use data and the Johns Hopkins ACG® System Aggregated Diagnosis Groups (ADGs) case-mix adjustment system (version 10). At least one major ADG categorized as:   - time-limited major - chronic medical, unstable - psychosocial, unstable - progressive or likely to recur - malignancy | By the end of the first year after arrival |
| **COVARIATES** | | | |
| Age at the time of arrival | Registered Persons Database (RPDB) (healthcare registry) | Mean age +/-SD  Median (IQR)  Age group: 0-5, 6-11, 12-17, 18-30, 31-45, 46-65, 65+ | On the arrival date |
| Sex | Registered Persons Database (RPDB) (healthcare registry) | Sex (male/female) of the individual as recorded in the individual’s health card. We recognize that this binary categorization does not necessarily reflect gender identity. | Health care registration |
| Census area-level material deprivation quintile | Ontario Marginalization index (ON-MARG) | Based on the residential postal code recorded in the RPDB as of OHIP registration and the Statistics Canada census of 2016.  Categorized into Q1 [least deprived], Q2, Q3, Q4 and Q5 [most deprived].  Those areas with suppressed deprivation index data were merged with the most marginalized, as they are usually low income. | Health care registration |
| Rurality | Registered Persons Database (RPDB) | Based on the residential postal code recorded in the RPDB as of OHIP registration and the Statistics Canada census of 2016.   - Rural, urban, missing | Health care registration |
| Canadian language ability at arrival | Immigration, Refugees, and Citizenship Canada’s (IRCC) Permanent Resident Database | Self-reported for resettled refugees   - Bilingual, English, French, None - Missing data merged with None | Time of immigration application |
| World region of birth | Immigration, Refugees, and Citizenship Canada’s (IRCC) Permanent Resident Database | Based on country of citizenship.   - Africa & Middle East - Americas - Asia & Pacific - Europe - Stateless - USA - Not States | Time of immigration application |
| Secondary migration | Immigration, Refugees, and Citizenship Canada’s (IRCC) Permanent Resident Database | A person was considered a secondary migrant when their country of last permanent residence differed from their country of birth. | Time of immigration application |
| Marital status at arrival | Immigration, Refugees, and Citizenship Canada’s (IRCC) Permanent Resident Database | Self-reported for resettled refugees   - Single, married, separated/divorced/widowed - Missing data were merged with separated/divorced/widowed | Time of immigration application |
| Highest education level at arrival | Immigration, Refugees, and Citizenship Canada’s (IRCC) Permanent Resident Database | Self-reported for resettled refugees   - ≤ secondary, trade/diploma/some university, ≥ Bachelors degree - Missing data were merged with ≤ secondary. | Time of immigration application |
| Family status | Immigration, Refugees, and Citizenship Canada’s (IRCC) Permanent Resident Database | - Principal applicant, spouse or common-law partner, child or other dependent | Time of immigration application |
| Drive Time to CHC (minutes) | Registered Persons Database (RPDB) (healthcare registry) | Based on residence recorded in the healthcare registry. Driving time by car at 40 km/hour with no impediments.   - Quantiles: ≤3, 3 – 10, and >10 minutes) - Missing data were excluded from CHC related analyses. | At the end of the first year after arrival |
