# Supplementary material for: Primary health care utilization in the first year after arrival by refugee sponsorship model in Ontario, Canada: A population-based cohort study
Source: PLoS One. 2023 Jul 26;18(7):e0287437. doi: 10.1371/journal.pone.0287437 (PMC10370760; doi:10.1371/journal.pone.0287437)
Supplement: S3 Table — (DOCX) [file pone.0287437.s004.docx]

# S3 Table: Dataset creation and analysis plan

| Project Cohort | | |
| --- | --- | --- |
| **Study Design** | Cohort study  Matched cohort study  Case-control study  Cross-sectional study  Other (specify): | |
| **Index Event / Inclusion Criteria** | - All Resettled refugees that landed in Ontario from April 1, 2008 to March 31, 2017. These groups are defined using the CIC/IRCC var CATEG/IMMIGRANT_CATEGORY - The **index date** for inclusion in the study cohort is the landing date (CIC/IRCC var LANDING_DATE) | |
| **Estimated Size of Cohort**  **(if known)** | 59,912 | |
| **Exclusions (in order)** | *Step* | Description |
|  | 1 | Invalid IKN OR IKN not found in RPDB |
|  | 2 | Missing age in RPDB |
|  | 3 | Missing sex in RPDB |
|  | 4 | Death prior to or on index date |
|  | 5 | Any CIC record prior to April 1, 2008 |
|  | 6 | Age ≥ 110 at beginning of accrual window (April 1, 2008) |

| Project Time Frame Definitions | | |
| --- | --- | --- |
| **Resettled refugees landed in Ontario**  CIC: April 1, 1985 to Index date  **Look-back Window**  Up to 1 year from and including index date (March 31, 2018)  **Observation Window**  (in which to look for outcomes)  Landing date  **Index Event Date**  April 1, 2008 to March 31, 2017  **Accrual Window**  March 31, 2018  **Max Follow-up Date** | |  |
| **Accrual Start/End Dates** | April 1, 2008 to March 31, 2017 |  |
| **Max Follow-up Date** | March 31, 2018 |  |
| **When does observation window terminate?** | Follow subjects forward from the index date to the first of the following censoring events:   - Death date - 1 year from index date, including index date |  |
| **Lookback Window(s)** | - Apr 1, 1985 for CIC records |  |

| Variable Definitions (add additional rows as needed) | | |
| --- | --- | --- |
| **Primary Exposures** | **The two primary exposures are defined as follows:**   1. **Era of landing (CIC/IRCC)**  - Using the CIC/IRCC var LANDING_DATE, create the following categories:  1. **Pre-Syrian Era –** All individuals with a landing date from April 1, 2008 through to October 31, 2015 2. **Syrian Era -** All individuals with a landing date from November 1, 2015 through to March 31, 2017 3. **Sponsorship Model (CIC/IRCC)**  - Using CIC_IRCC variable IMMIGRANT_CATEGORY, create the following categories:  1. **Privately sponsored refuges (PSRs)** 2. **Government-assisted refugees (GARs)** 3. **Blended Visa Office-reffered Refugees (BVORs)**  - **Note:** For those who arrived in the **Pre-Syrian era only,** combine BVORs with PSRs as an aggregated PSR category.   **Stratify the cohort by both primary exposures into the following five groups for the primary analyses:**   - - Pre-Syrian Era PSRs   - Pre-Syrian Era GARs   - Syrian Era PSRs   - Syrian Era BVORs   - Syrian GARs |  |
| **Secondary Exposures** | **Country cohort/country of origina (CIC, IRCC)**   - Country cohort groups are **mutually exclusive**, follow steps in order below: - First identify individuals in the following Special Programs (CIC/IRCC var SPEC_P/SPECIAL_PROGRAM) and set them each aside from the rest of the cohort:   - UE1 - Syria Voluntary Evacuation = 15473   - Rohingyas = 357/ ROH – Rohingyas=15461   - Refugees In Mae La Oon Camp, Thailand=347/ MLO - Myanmar Refugees in La Oon Camp, Thailand=15453   - Bhutanese Refugees In Nepal=356/BHU - Bhutanese Refugees in Nepal=15460 - Proceed to create the country cohort group, starting with ‘**a)** **Syrian refugees in the Syrian era’**:  1. **Syrian refugees in the Syrian era**     - Restrict to individuals with an index date between Nov 1, 2015 and Mar 31, 2016    - Identify all individuals with the IRCC Syrian Refugee flag for Wave 1 or Wave 2      - IRCC var Syrian_refugee_flag =1,2    - Aggregrate with remaining individuals in the Syrian Refugee Processing Special program  - IRCC var SPECIAL_PROGRAM 🡪 SRP - Syrian Refugee Processing = 15528  1. **Iraq**     - Include all individuals in the Syrian Voluntary Evacuation special program  - IRCC var SPECIAL_PROGRAM 🡪 UE1 - Syria Voluntary Evacuation = 15473   - Aggregate with individuals with Iraq Country of Citizenship     - CIC/IRCC vars FCITZ/COUNTRY_CITIZENSHIP=224/2006  1. **Afghanistan**     - Identify individuals with Afghanistan Country of Citizenship      - CIC/IRCC vars FCITZ/COUNTRY_CITIZENSHIP=252/2014 2. **Iran**     - Identify individuals with Iran Country of Citizenship      - CIC/IRCC vars FCITZ/COUNTRY_CITIZENSHIP=223/2005 3. **Somalia**     - Identify individuals with Somalia Country of Citizenship      - CIC/IRCC vars FCITZ/COUNTRY_CITIZENSHIP=182/1982 4. **Eritrea**    - Identify individuals with Eritrea Country of Citizenship      - CIC/IRCC vars FCITZ/COUNTRY_CITIZENSHIP=162/1963 5. **Congo**    - Identify individuals with Congo Country of Citizenship      - CIC/IRCC vars FCITZ/COUNTRY_CITIZENSHIP=162/1963 6. **Myanmar**    - Include all individuals in the Rohingyas special program    - CIC/IRCC var SPEC_P/SPECIAL_PROGRAM 🡪 Rohingyas = 357/ ROH – Rohingyas=15461    - Include all individuals in the Rohingyas special program    - CIC/IRCC var SPEC_P/SPECIAL_PROGRAM 🡪 Refugees In Mae La Oon Camp, Thailand=347/ MLO - Myanmar Refugees in La Oon Camp, Thailand=15453    - Aggregate with individuals with Myanmar Country of Citizenship   CIC/IRCC vars FCITZ/COUNTRY_CITIZENSHIP=241/2011   1. **Bhutan**    - Include all individuals in the Rohingyas special program    - CIC/IRCC var SPEC_P/ SPECIAL_PROGRAM 🡪 Bhutanese Refugees In Nepal=356/BHU - Bhutanese Refugees in Nepal=15460    - Aggregate with individuals with Bhutan Country of Citizenship   CIC/IRCC vars FCITZ/COUNTRY_CITIZENSHIP=254/2016   1. **Ethiopia**    - Identify individuals with Ethiopia Country of Citizenship      - CIC/IRCC vars FCITZ/COUNTRY_CITIZENSHIP=161/1962 2. **All other African countries:**     - Identify remaining individuals with Region2 = Africa 3. **Other:** All individuals not captured above |  |
| **Baseline Characteristics** | The following socio demographic characteristics at landing date will be captured (CIC/IRCC, RPDB):   - Age (numerical), derived from CIC/IRCC var LANDING_DATE subtracted from RPDB var BDATE (Date of birth)   - mean ± SD; median (IQR) - Age (categorical), n(%) - 0-5 - 6-11 - 12-17 - 18-30 - 31-45 - 46-65 - >65 - Sex, from RPDB var SEX, n(%) - Male - Female - ONMARG material deprivation (deprivation_q_da) index score per person (categorical), n(%) - 2 (Least marginalized) to 5 (Most marginalized) - Note: Those with a score of less than 2 were grouped with least marginalized and suppressed data was merged with the most marginalized - Rurality (%getdemo), n(%)   - Missing   - Urban   - Rural - Canadian language ability (CIC/IRCC var CAN_LANG/OFFICIAL_LANGUAGE)   - Bilingual     - 3=Both French and English/15222=Bilingual   - English     - 1=English/15220=English   - French     - 2=French/15221=French   - None (includes missing)     - 4=Neither/15223=None - World Region – Broad Categories (CIC/IRCC var world_area_eng_desc) and Detailed Categories (CIC/IRCC var world_area_region_eng_desc) - Africa & Middle East   - Africa Unspecified   - Central Africa   - East Africa   - Middle East   - North Africa   - Southern Africa   - Western Africa - Americas   - Caribbean   - Central America   - South America   - Western Hemis. (Others) - Asia & Pacific   - Australasia   - East Asia   - Oceania & Asia Unspecified   - South Asia   - Southeast Asia - Canada   - Canada - Europe   - Eastern Europe   - Europe Unspecified   - Northern Europe   - Southern Europe   - United Kingdom   - USSR (former)   - Western Europe   - Yugoslavia (former) - North America   - United States of America - Stateless   - Stateless - Not Stated   - Not Stated - Syrian Refugee Status – Use the definition for ‘Country Cohort a) Syrian refugees in the Syrian era’, in Secfondary Exposure above. - Yes - No - Marital status (limit to individuals aged 18 years and older), from CIC/IRCC var M_STAT/MARITAL_STATUS - Single   - 1=Single/8871=Single - Married or common-law   - 2=Married/8872=Married; 6=Common law/8876=Common law - Separated, divorced, or widowed (includes missing)   - 3=Widowed/8873=Widowed; 4=Divorced/8874=Divorced; 5=Separated/8875=Separated; 7=Annulled marriage/8877=Annulled marriage - Highest education level – Broad Categories (limit to individuals aged 25 years and older), from CIC/IRCC var FEDUC/EDUCATION_QUALIFICATION - Secondary or less (includes missing)   - Value missing or -1=Not stated; 0=None/7668=None; or 1=Secondary or less/7660=Secondary or less - Trade or Diploma, or some University (less than Bachelor’s)   - 4=Some University - No Degree/7663=Some University - No Degree; 2=Formal Trade Cert. or Apprenticeship/7661= Formal Trade Cert. or Apprenticeship; or 3=Non-University Certificate or Diploma/7662=Non-University Certificate or Diploma - Bachelor’s or higher   - 5=Bachelor’s/7664=Bachelor’s; 6=Some Post-Grad. Education - No Degree/7665=Some Post-Grad. Education - No Degree; or 7=Master's Degree/7666=Master's Degree; 8=Doctorate/7667=Doctorate - Secondary migration prior to arrival, defined as those who have a different Country of Citizenship from their Country of Last Permanent Residence, from CIC/IRCC vars FCITZ/COUNTRY_CITIZENSHIP and FCLPR/COUNTRY_RESIDENCE. - Yes (Country of Citizenship ≠ Country of Residence) - No (Country of Citizenship = Country of Residence, or at least one is missing) - Family Status, from CIC/IRCC var FSAT/FAMILY_STATUS - Principal applicant   - 1=Principal Applicant/7890=Principal Applicant - Spouse or common-law partner   - 2=Spouse/7891=Spouse; 5=Common - law partner/7894=Common Law - Child or other dependent (including missing)   - 3=Dependent of a Principal Applicant Excluding Post 1991 J88 Applications/7892=Dependant of a Principal Applicant Excluding Post 1991 J88 Applications; 4=J88 Dependent/7893=J88 Dependant; 1000001=Dependants; 7899=Adopted Child; 56013=Adoptive Parent; 7895=Child; 7896=Step-Child; 7897=Grandchild; 56012=Step-Grandchild; 7898=Other; 56011=Parent; 56014=Unaccompanied Minors - **Driving distance to CHC (GIS network analysis, patient postal codes)** - In minutes: ≤3, 3 – 10, and >10 - Use GIS network analysis showing the access distances and times from patient postal codes to the nearest CHC based on driving at the speed limits posted along existing roads |  |
| **Healthcare use** **indicators of ‘positive’ settlement (Primary & Secondary Outcomes):** | ***Primary Outcomes:***  **Time (days) from landing date to first PC healthcare contact during year 1 - includes any PC visit to a GP, Pediatrician, or NP; or any visit to a GP or NP at a CHC (data sources: OHIP and CHC)**   - Calculate number of days (mean±SD; median, IQR) between landing date (CIC/IRCC var LANDING_DATE) and the first of any of the following visit types:   - outpatient (OHIP LOCATION=O,H,L) PC visit for any PC OHIP claim appearing anywhere among all of their feecodes per day (OHIP SPEC=00, 26, and 76, and see Appendix A for OHIP PC visit fee codes)   - any visit to a physician or nurse practitioner at a CHC   **Individuals with any PC visits during the first 2 months - includes any visit to a GP, Pediatrician, or NP; or any visit to a GP or NP at a CHC (data sources: OHIP, CHC)**   - n (% ) of subjects with at least one outpatient (OHIP LOCATION=O,H,L) PC visit for any PC OHIP claim appearing anywhere among all of their feecodes per day (OHIP SPEC=00, 26, and 76 and see Appendix A for OHIP PC visit fee codes) OR any visit to a CHC in the first 2 months (will describe by provider – GP/NP) - Number of PC visits per person (mean ± SD; median (IQR)) in the first 2 months   **PC visits during year 1 - includes any visit to a GP, Pediatrician, or NP; or any visit to a GP or NP at a CHC (data sources: OHIP and CHC)**   - n (% ) of subjects with at least one outpatient (OHIP LOCATION=O,H,L) PC visit for any PC OHIP claim appearing anywhere among all of their feecodes per day [OHIP SPEC=00, 26, 76 and see Appendix A for OHIP PC visit fee codes] OR any visit to a CHC in the first year - Number of PC visits per person (mean ± SD; median (IQR))   **PC affiliation at the end of year 1 (assignment as follows)**   - Care in CHC:   - Create flags by linking IKN to CHC data for servdates between April 1, 2008 and May 31, 2017   - If any CHC visit in the first year following OHIP eligibility, assign to CHC as PC affiliation (see list of categories below). - Check in CAPE to see if individual is rostered to a physician in a primary care enrolment model (CAPE var PROGTYPE), if yes, then describe the enrolment model (PEM) of the physician - For all others, take all PC OHIP claims appearing anywhere among all of their feecodes per day [see Appendix A for OHIP PC visit fee codes] for the past year (restrict to OHIP SPEC=00 [GP/FP] or 26 [paed] or 76 (NP) - Assign the provider as the one who provides highest dollar values of all PC visits; if tie, assign the one who provided the most recent visit; - Link OHIP PHYSNUM to IPDB as assign physician specialty (GP/FP – no PC model; paediatrician; or nurse practitioner), restrict to where mainspecialty is:   - GP/FP, includes mainspecialty:     - GP/FP     - FP/emergency medicine     - community health/public health   - Paediatrician:     - Pediatrics/Paediatrics   - Nurse practitioner - If no PC visit in past year, then assign ‘no PC visit in previous year/no PCP’; - Describe PC affiliation using the following PEM categories:   - Immigrant-focused Community Health Centre [see Appendix B for list of these CHCs. The definition includes any Ontario CHCs where >25% of clients aged older than 10 years who visited the CHC between April 1, 2013 and March 31, 2015 were ‘newcomers’ to Ontario who first became eligible for OHIP within the 10 years prior to their CHC visit and who did not die on or before March 31, 2015 AND/OR CHCs which receive specific funding for and have an organizational mandate to provide specialized programs for refugees and immigrants]   - Other CHC or missing name of CHC   - Comprehensive PC model (all other PROGTYPE, or paediatrician)   - Other (walk-ins, solo-practitioners, no PC model)   - No PCP (no PC visit in the last year)   **Community Health Centre (CHC) visits during year 1 (CHC)**   - n (% ) of subjects with at least one CHC visit - Number of CHC visits per person (mean ± sd, median (IQR)) - Any CHC visit stratified by healthcare provider type (GP, NP), n (%) - Any CHC visit to a CHC specializing in refugee and immigrant health. See Appendix B for a list of these CHCs. Note: We defined CHCs specializing in refugee and immigrant health as those CHCs which met **either or both** of the following criteria:   - >25% of clients aged older than 10 years who visited the CHC between April 1, 2013 and March 31, 2015 were ‘newcomers’ to Ontario who first became eligible for OHIP within the 10 years prior to their CHC visit and who did not die on or before March 31, 2015   - Receive specific funding for and have an organizational mandate to provide specialized programs for refugees and immigrants   **Specialist visits during year 1 (OHIP, IPDB)**   - n (%) of subjects with at least one outpatient (OHIP LOCATION=O,H,L) specialist visit in the first year. Link OHIP PHYSNUM to IPDB to determine physician MAINSPECIALTY for all non-PC visits for any OHIP feecodes **except the PC visits feecodes in Appendix A** **AND excluding OHIP SPEC=00**   **Emergency department (ED) visits during year 1 (NACRS)**   - Note: Exclude transfers from another ED (exclude from_type= ‘E’; DEDUP= ‘T’ in %getnacrs - Categorize ED visits by CTAS score:   - Low acuity= CTAS 1-3   - High acuity= CTAS 4-5   - Calculate n(%) with a high acuity visit, n(%) with a low acuity visit   **Hospitalizations during year 1 (DAD, SDS)**   - N (%) of hospitalizations in the past year (including any pregnancy-related, planned, or same day surgeries).   **Death (RPDB)**   - n(%) of subjects who died during follow-up   ***Secondary Outcomes:***  **Time (days) from landing date to first outpatient healthcare contact during year 1 - includes any Primary Care (PC) visit to a General Practitioner (GP), Pediatrician, or Nurse Practitioner (NP); any visit to a GP or NP at a Community Health Centre (CHC); Specialist visit; or emergency department visit (data sources: OHIP, CHC, IPDB, and NACRS)**   - Calculate number of days (mean±SD; median, IQR) between landing date (CIC/IRCC var LANDING_DATE) and the first of any of the following visit types:   - outpatient (OHIP LOCATION=O,H,L) PC visit for any PC OHIP claim appearing anywhere among all of their feecodes per day (OHIP SPEC=00, 26, and 76, and see Appendix A for OHIP PC visit fee codes)   - outpatient (OHIP LOCATION=O,H,L) specialist visit. Link OHIP PHYSNUM to IPDB to determine physician MAINSPECIALTY for all non-PC visits for any OHIP feecodes **except the PC visits feecodes in Appendix A and excluding OHIP SPEC=00**   - any visit to a physician or nurse practitioner at a CHC   - any ED visit (planned or unplanned) - Note: Exclude transfers from another ED (exclude from_type= ‘E’; DEDUP= ‘T’ in %getnacrs) - Stratify the total number of outpatient healthcare visits on and individual’s first day of healthcare contact by the following mutually exclusive categories:   - n (%) healthcare contacts on an individuals’ day of first contact which were PC visits to a GP, Pediatrician, or NP (NOT including CHC visits)   - n (%) healthcare contacts on an individuals’ day of first contact which were CHC visits   - n (%) healthcare contacts on an individuals’ day of first contact which were specialist visits   - n (%) healthcare contacts on an individuals’ day of first contact which were ED visits   **Individuals with any outpatient healthcare visit during year 1 - includes any PC visit to a GP, Pediatrician, or NP; any visit to a GP or NP at a CHC; Specialist visit; or emergency department visit (data sources: OHIP, CHC, IPDB, and NACRS)**   - n (% ) of subjects with at least one outpatient visit   **NP visits during year 1 – does not include NP visits at CHCs (data sources: OHIP)**   - n (% ) of subjects with at least one outpatient (OHIP LOCATION=O,H,L) PC visit for any PC OHIP claim appearing anywhere among all of their feecodes per day [OHIP SPEC=76 and see Appendix A for OHIP PC visit fee codes] - Number of NP visits per person (mean ± SD; median (IQR))   **Community Health Centre (CHC) visits during year 1 (CHC)**   - See detailed methodology in primary outcome above - Any CHC visit stratified by healthcare provider type (GP, NP), n (%)   **Specialist visits during year 1 (OHIP, IPDB)**   - See detailed methodology in primary outcome above - Number of specialist visits per person (mean ± sd, median (IQR))   **Emergency department (ED) visits during year 1 (NACRS)**   - Note: Exclude transfers from another ED (exclude from_type= ‘E’; DEDUP= ‘T’ in %getnacrs - Any ED visit (planned and unplanned), n(%) - Number of ED visits per person (mean ± SD, median (IQR)) - Categorize ED visits by CTAS score:   - Low acuity= CTAS 1-3   - High acuity= CTAS 4-5   - Calculate rate of ED visits by acuity, mean (SD), median (IQR) - n(%) of **all ED visits** where the patient was transported via ambulance, includes visits with the following values of NACRS var ADMAMBUL:   - A = Air Ambulance; C = Combination of ground and air ambulance; G = Ground ambulance; and W = Water Ambulance - n(%) of **all ED visits** where the patient was transported via ambulance, stratified by low acuity vs high acuity visits   **Hospitalizations during year 1 (DAD, SDS)**   - Hospitalizations in the past year (including any pregnancy-related, planned, or same day surgeries). - Number of hospitalizations per person (mean ± sd, median (IQR)) |  |

| **Analysis Plan** |
| --- |
| **Cohort creation**   - - Identify all refugees and immigrant (primary exposure categories 1-4.2) with a landing date (LANDING_DATE) in Ontario between April 1, 2008 and May 31, 2016 AND OHIP eligibility start date (SRTELIG) in the same period. Use landing date as the index date to anchor all analyses on   - If more than one CIC record exists during the accrual period, use the most recent record.   - Prepare a flowchart of the cohort creation (Appendix Figure 1)   **Baseline covariates**   - Generate baseline covariates as described above across sponsorship models, stratified by pre-Syrian and Syrian era (Table 1) - Use the following statistical analyses depending on the type of baseline covariate:   - The mean +/- s.d., (or mean with 95% Confidence Interval), and the P-value from a one-way ANOVA   - The median and interquartile range (IQR, i.e. the 25th and 75th percentile), and the P-value from a Kruskal-Wallis test.   - For categorical variables: The N and %, and the p-value from a chi-squared.   - For binary 0/1 variables: The N and % for the value "1", and the p-value from a chi-squared. - Missing values will be treated as follows: Canadian language ability merged with none, marital status merged with separated, divorced or widowed, and education merged with secondary or less - Missing CHC driving distances were be excluded from all analyses due to lack of postal code information   **Outcomes**  Follow the methodology outlined above for generation of each primary and secondary outcome variable and corresponding statistics   - Generate a table of healthcure utilization (visits to PC and CHCs), as well as other healthcare use variable across sponsorship models, stratified by pre-Syrian and Syrian era (Table 2 and Appendix Table 1)   - Use the following statistical analyses depending on the type of outcome variable and statistics we are interested in:     - The mean +/- s.d., (or mean with 95% Confidence Interval), and the P-value from a one-way ANOVA     - The median and interquartile range (IQR, i.e. the 25th and 75th percentile), and the P-value from a Kruskal-Wallis test.     - For categorical variables: The N and %, and the p-value from a chi-squared.     - For binary 0/1 variables: The N and % for the value "1", and the p-value from a chi-squared. - Use Kaplan-Meier survival curves to plot the likelihood of a PC visit over time by settlement model and era within the first year of landing date (Figure 1) - Evaluate time to first PC visit and any CHC visits within the first year of landing by settlement model and era (Table 3):   - For both outcomes below, we have a pre-specified hypothesis of an interaction between settlement model and era     - Test this with an interaction term and if statistically significant, then we will re-parameterize the exposure to account for both era and sponsorship model   - Cox proportional hazards models should be used to test the association between time to first PC visit by sponsorship model and era of landing   - Logistic regression should be used to model the outcome of any CHC visit in the first year after landing by sponsorship model and era of landing   - Models will be adjusted for age, sex, deprivation index, urban living, Canadian language ability, world region, secondary immigration, season of arrival (time to PC visit only) and time to travel to CHC (for CHC model only)   - Models will only be generated among those living in urban areas - Secondary analysis: Evaluate time to first PC visit and any CHC visits within the first year of landing by country of origina and era (Table 4):   - Use same analytic plan as outlined above (including testing for a significant interaction between sponsorship model and country of origin), however, additionally adjust for year of landing. - Subgroup analysis: Among those aged ≥25, additionally adjust for marital status and education to test their impact on sponsorship model and era (Appendix Table 2) - Post-hoc analysis: explore the characteristics of individuals: 1) who had a PC visit in the first year compared to those who did not and 2) among those with no PC visits by era (Appendix Table 3)   - Observe differences in baseline covariates and health care use among PSRs only as results showed they have fewer PC visits than GARs |
