# Supplementary material for: Primary health care utilization in the first year after arrival by refugee sponsorship model in Ontario, Canada: A population-based cohort study
Source: PLoS One. 2023 Jul 26;18(7):e0287437. doi: 10.1371/journal.pone.0287437 (PMC10370760; doi:10.1371/journal.pone.0287437)
Supplement: S4 Table — (DOCX) [file pone.0287437.s005.docx]

# S4 Table: List of all primary care (PC) visit feecodes

| **Category** |  | **Feecode** | **Description** |
| --- | --- | --- | --- |
| 19 Primary Care Codes |  | A001 | MINOR ASSESS.-F.P./G.P. |
|  |  | A002 | 18 MONTH WELL BABY CHECK -GP/FP |
|  |  | A003 | GEN. ASSESS. -F.P./G.P. |
|  |  | A007 | INTERMED.ASSESS/WELL BABY CARE-F.P./G.P./PAED. |
|  |  | A903 | GEN/FAM PRACT-PRE-DENTAL/OPER.ASSESS LIMIT 2 PER YEAR/PT |
|  |  | E075 | GERIATRIC GENERAL ASSESSMENT PREMIUM |
|  |  | G212 | D./T. PROC.-ALLERGY-HYPOSENSITIZATION INJECTION PLUS BASIC |
|  |  | G271 | D./T. PROC.-CARDIOV.-ANTICOAGULANT SUPERVISION |
|  |  | G372 | D./T. PROC.-INJECTIONS-INTRADERMAL/MUSCULAR ETC. EA. ADD. |
|  |  | G373 | D./T. PROC.-INJ. INTRADERMAL/MUSC. BASIC FEE (SHICK TEST) |
|  |  | G365 | D./T. PROC.-GYNAECOLOGY-PAPANICOLAOU SMEAR |
|  |  | G538 | D&T IMMUNIZATION-WITH VISIT, EACH INJECT. |
|  |  | G539 | D&T IMMUNIZATION-SOLE REASON,FIRST INJECTION |
|  |  | G590 | INFLUENZA AGENT +VISIT |
|  |  | G591 | INFLUENZA AGENT SOLE REASON |
|  |  | K005 | INDIVIDUAL CARE PER 1/2 HR |
|  |  | K013 | COUNSELLING-ONE OR MORE PEOPLE-PER 1/2HR. |
|  |  | K017 | ANNUAL HEALTH EXAM-CHILD AFT. 2ND BIRTHDAY. |
|  |  | P004 | OBS.-PRENATAL CARE-MINOR PRENATAL ASSESS.-SUBSEQ.PRENAT.VIS. |
| Paediatric codes |  | A261 | MINOR ASSESS.-PAED. |
|  |  | A268 | 18 MONTH WELL BABY CHECK - PAEDS |
|  |  | K267 | ANNUAL HEALTH EXAM-CHILD-AFT. 2ND BIRTHDAY PAED. |
|  |  | K269 | ANNUAL HEALTH EXAM-PAEDIATRICS-ADOLESCENT-OFFICE |
| Recent Additions   Note* These codes start in 2013 |  | K130 | Periodic health visit - adolescent |
|  |  | K131 | Periodic health visit - adult aged 18 to 64 inclusive |
|  |  | K132 | Periodic health visit - adult 65 years of age and older |
