# Supplementary material for: Primary health care utilization in the first year after arrival by refugee sponsorship model in Ontario, Canada: A population-based cohort study
Source: PLoS One. 2023 Jul 26;18(7):e0287437. doi: 10.1371/journal.pone.0287437 (PMC10370760; doi:10.1371/journal.pone.0287437)
Supplement: S5 Table — (DOCX) [file pone.0287437.s006.docx]

# S5 Table: List of all Ontario Community Health Centres (CHCs), as well as indicators for those who specialize in care for immigrants/refugees and those who are located in high immigrant/refugee areas.

| CHC Name | Specialize in care for immigrants/refugees | Located in high immigrant/refugee areas |
| --- | --- | --- |
| Access Alliance Multicultural | Y | N |
| Centre Francophone de Toronto | N | Y |
| Women's Health in Women's Hands | Y | N |
| Wellfort | Y | N |
| Rexdale | Y | N |
| Scarborough | Y | N |
| Unison | N | Y |
| Black Creek | Y | N |
| Stonegate | Y | N |
| South-East Ottawa | N | Y |
| CSC Hamilton Welland | N | Y |
| Flemingdon Health Centre | N | N |
| Vaughan | N | N |
| TAIBU | N | Y |
| Parkdale | N | Y |
| Carlington | N | Y |
| Davenport-Perth | N | N |
| Somerset West | N | Y |
| Hamilton Urban Core | N | N |
| Pinecrest-Queensway | N | Y |
| Kitchener Downtown | N | N |
| LAMP | N | Y |
| North Hamilton | N | N |
| Centretown | N | Y |
| South Riverdale | N | N |
| East End | N | N |
| Planned Parenthood | N | N |
| Four Villages | N | N |
| London | N | N |
| Guelph | N | N |
| Regent Park | N | N |
| Central Toronto Queen West | N | N |
| Seaway Valley | N | N |
| Niagara Falls Niagara Falls (HNHB | N | N |
| CSC de l'Estrie | N | N |
| Windsor Essex | N | N |
| Anishnawbe Health Toronto | N | N |
| Sandy Hill | N | N |
| Belleville | N | N |
| Rainbow Valley | N | N |
| City Centre Health | N | N |
| Bridges | N | N |
| Central St Thomas | N | N |
| Quest | N | N |
| Langs Farm | N | N |
| Anne Johnston Health Station | N | N |
| NorWest | N | N |
| Grand River | N | N |
| Mary Berglund | N | N |
| Kingston Kingston | N | N |
| CAREA | N | N |
| Woodstock | N | N |
| Chatham Kent | N | N |
| Port Hope | N | N |
| Chigamik Midland (NSM | N | N |
| North Lambton | N | N |
| North Lanark | N | N |
| CSC du Témiskaming | N | N |
| Woolwich | N | N |
| Brock CHC | N | N |
| West Nippising | N | N |
| South East Grey | N | N |
| Country Roads | N | N |
| Kawartha Lakes | N | N |
| Rideau | N | N |
| CSC de Grand Sudbury | N | N |
| Kapuskasing | N | N |
| CSC de Sudbury-Est | N | N |
| Gateway | N | N |
| Misiway Milopemahtesewin | N | N |
| West Elgin | N | N |
| Grand Bend | N | N |
