# Supplementary material for: Primary health care utilization in the first year after arrival by refugee sponsorship model in Ontario, Canada: A population-based cohort study
Source: PLoS One. 2023 Jul 26;18(7):e0287437. doi: 10.1371/journal.pone.0287437 (PMC10370760; doi:10.1371/journal.pone.0287437)
Supplement: S6 Table — (DOCX) [file pone.0287437.s007.docx]

# S6 Table. Other healthcare use in the first year among resettled refugees that landed in Ontario between April 1, 2008 and March 31, 2017, by era of landing and sponsorship model.

|  | **Pre-Syrian era – April 1, 2008 to October 31, 2015** | | | | **Syrian era – November 1, 2015 to March 31, 2017** | | | | |
| --- | --- | --- | --- | --- | --- | --- | --- | --- | --- |
| Sponsorship Model | Government-assisted refugees (GARs) | Privately sponsored refugees (PSRs)* | Total | P-value | Government-assisted refugees (GARs) | Blended Visa Office-referred refugees (BVORs) | Privately sponsored refugees (PSRs) | Total | P-value |
| **Cohort size, N** | 17,623 | 16,968 | 34,591 |  | 12,051 | 2,695 | 10,011 | 24,757 |  |
| **Visits to Primary Care (PC) and Community Health Centres (CHCs)** | | | | |  |  |  |  |  |
| **Days from landing date to first outpatient healthcare contact** ^a,b,c,d^ | | | | |  |  |  |  |  |
| Mean ± SD | 70.7 ± 71.2 | 146.4 ± 84.9 | 104.9 ± 86.3 | <.001 | 65.4 ± 60.0 | 69.4 ± 72.5 | 111.6 ± 87.9 | 83.4 ± 76.4 | <.001 |
| Median (IQR) | 47 (22-93) | 129 (93-196) | 87 (35-149) | <.001 | 49 (25-85) | 43 (19-95) | 93 (40-158) | 59 (29-112) | <.001 |
| **Any outpatient healthcare visit N (%)** ^a,b,c,d^ | | | | |  |  |  |  |  |
|  | 16,477 (93.5%) | 13,604 (80.2%) | 30,081 (87.0%) | <.001 | 11,714 (97.2%) | 2,513 (93.2%) | 8,735 (87.3%) | 22,962 (92.7%) | <.001 |
| **Types of outpatient healthcare visit, N (%)** ^e^ | | | | |  |  |  |  |  |
| PC visits to GP, pediatrician or NP | 12,275 (69.7%) | 11,695 (68.9%) | 23,970 (69.3%) | 0.14 | 7,445 (61.8%) | 1,614 (59.9%) | 7,071 (70.6%) | 16,130 (65.2%) | <.001 |
| CHC visits to GP or NP | 1,662 (9.4%) | 364 (2.1%) | 2,026 (5.9%) | <.001 | 2,557 (21.2%) | 506 (18.8%) | 396 (4.0%) | 3,459 (14.0%) | <.001 |
| Specialist visits | 1,849 (10.5%) | 878 (5.2%) | 2,727 (7.9%) | <.001 | 1,262 (10.5%) | 240 (8.9%) | 862 (8.6%) | 2,364 (9.5%) | <.001 |
| ED | 859 (4.9%) | 799 (4.7%) | 1,658 (4.8%) | 0.47 | 571 (4.7%) | 169 (6.3%) | 478 (4.8%) | 1,218 (4.9%) | 0.003 |
| **Any PC visit to a NP, N (%)** | 1,777 (10.1%) | 315 (1.9%) | 2,092 (6.0%) | <.001 | 2,309 (19.2%) | 395 (14.7%) | 368 (3.7%) | 3,072 (12.4%) | <.001 |
| **Number of PC visits per person to a NP** | | | |  |  |  |  |  |  |
| Mean ± SD | 3.20 ± 2.93 | 3.38 ± 2.77 | 3.23 ± 2.91 |  | 3.08 ± 2.93 | 4.47 ± 3.99 | 3.83 ± 3.57 | 3.35 ± 3.20 | <.001 |
| Median (IQR) | 2 (1-4) | 3 (1-5) | 2 (1-4) |  | 2 (1-4) | 3 (1-6) | 3 (1-5) | 2 (1-4) | <.001 |
| **Any CHC visit to a GP N (%)** | | | | |  |  |  |  |  |
|  | 1,688 (9.6%) | 290 (1.7%) | 1,978 (5.7%) | <.001 | 2,286 (19.0%) | 354 (13.1%) | 306 (3.1%) | 2,946 (11.9%) | <.001 |
| **Any CHC visit to a NP N (%)** | |  |  |  |  |  |  |  |  |
|  | 1,777 (10.1%) | 314 (1.9%) | 2,091 (6.0%) | <.001 | 2,307 (19.1%) | 395 (14.7%) | 368 (3.7%) | 3,070 (12.4%) | <.001 |

^a^ Primary care (PC) visits to a general practitioner, pediatrician or nurse practitioner

^b^ PC visits to a GP or nurse at a Community Health Centre (CHC)

^c^ Specialist visits

^d^ Emergency department visits

^e^ may include multiple same-day visits per individua
