# Supplementary material for: Primary health care utilization in the first year after arrival by refugee sponsorship model in Ontario, Canada: A population-based cohort study
Source: PLoS One. 2023 Jul 26;18(7):e0287437. doi: 10.1371/journal.pone.0287437 (PMC10370760; doi:10.1371/journal.pone.0287437)
Supplement: S7 Table — (DOCX) [file pone.0287437.s008.docx]

# S7 Table. Cox proportional hazard ratios for the association between sponsorship model and era on time to first primary care (PC) visit and logistic odds ratio for the association between sponsorship era and era on any community health centre (CHC) visit in the first year of resettlement in all resettled refugees who landed in Ontario between April 1, 2008 and March 31, 2017.

|  | **Time to first PC visit (days)^1^ N=58,601** | | **Odds of CHC visit in year 1^2^ N=58,477** | |
| --- | --- | --- | --- | --- |
| **Covariate** | **Unadjusted HR (95% CI)** | **Adjusted HR (95% CI)** | **Unadjusted OR (95% CI)** | **Adjusted OR (95% CI)** |
| **Era of landing + Sponsorship model** |  |  |  |  |
| Syrian Era - Syrian GARs | 2.66 (2.59, 2.73) | 2.27 (2.19, 2.35) | 12.80 (11.55, 14.19) | 14.69 (12.98, 16.63) |
| Syrian Era - Syrian BVORs | 2.21 (2.11, 2.33) | 1.89 (1.79, 1.99) | 11.17 (9.71, 12.84) | 14.08 (12.05, 16.44) |
| Syrian Era - Syrian PSRs | 1.40 (1.36, 1.45) | 1.19 (1.14, 1.23) | 2.08 (1.81, 2.40) | 2.51 (2.15, 2.93) |
| Syrian Era - Non-Syrian GARs | 2.68 (2.56, 2.80) | 2.61 (2.49, 2.74) | 16.35 (14.39, 18.58) | 10.63 (9.27, 12.20) |
| Syrian Era - Non-Syrian BVORs | 1.81 (1.62, 2.01) | 1.76 (1.58, 1.97) | 11.27 (8.83, 14.39) | 6.66 (5.15, 8.61) |
| Syrian Era - Non-Syrian PSRs | 1.20 (1.15, 1.25) | 1.19 (1.14, 1.24) | 1.78 (1.49, 2.14) | 0.89 (0.73, 1.07) |
| Pre-Syrian Era GARs | 2.16 (2.11, 2.21) | 2.18 (2.12, 2.23) | 5.48 (4.95, 6.07) | 5.31 (4.77, 5.90) |
| Pre-Syrian Era PSRs (reference) | 1.00 | 1.00 | 1.00 | 1.00 |
| **Age group in years** |  |  |  |  |
| 0 to 5 |  | 1.22 (1.18, 1.25) |  | 0.91 (0.83, 0.99) |
| 6 to 11 |  | 1.00 (0.97, 1.03) |  | 0.88 (0.80, 0.96) |
| 12 to 17 |  | 0.93 (0.90, 0.96) |  | 0.86 (0.78, 0.95) |
| 18 to 30 (reference) |  | 1.00 |  | 1.00 |
| 31 to 45 |  | 1.21 (1.18, 1.24) |  | 1.16 (1.07, 1.25) |
| 46 to 65 |  | 1.42 (1.38, 1.47) |  | 1.21 (1.09, 1.34) |
| 66 to 100 |  | 1.62 (1.53, 1.71) |  | 1.24 (1.02, 1.50) |
| **Sex** |  |  |  |  |
| Female |  | 1.15 (1.13, 1.17) |  | 1.06 (1.01, 1.12) |
| Male (reference) |  | 1.00 |  | 1.00 |
| **Neighborhood Deprivation Quintile** |  |  |  |  |
| Q1 - least deprived |  | 1.06 (1.00, 1.13) |  | 0.96 (0.76, 1.20) |
| Q2 |  | 1.01 (0.97, 1.06) |  | 1.15 (0.98, 1.35) |
| Q3 |  | 1.02 (0.99, 1.06) |  | 1.09 (0.97, 1.22) |
| Q4 |  | 1.11 (1.09, 1.13) |  | 0.78 (0.73, 0.83) |
| Q5 - most deprived (reference)³ |  | 1.00 |  | 1.00 |
| **Canadian language ability** |  |  |  |  |
| English and/or French |  | 0.93 (0.91, 0.95) |  | 0.87 (0.82, 0.93) |
| None (Reference)⁴ |  | 1.00 |  | 1.00 |
| **World Region of Citizenship** |  |  |  |  |
| Africa |  | 1.05 (0.95, 1.15) |  | 0.95 (0.87, 1.05) |
| Americas |  | 0.94 (0.82, 1.07) |  | 2.05 (1.65, 2.56) |
| Asia & Pacific (reference) |  | 1.00 |  | 1.00 |
| Europe & USA |  | 1.20 (1.16, 1.23) |  | 0.64 (0.42, 0.98) |
| Middle East |  | 1.00 (0.97, 1.04) |  | 0.36 (0.32, 0.39) |
| Stateless⁴ |  | 0.92 (0.84, 1.01) |  | 1.13 (0.90, 1.43) |
| **Secondary migration** |  |  |  |  |
| Yes |  | 1.17 (1.14, 1.19) |  | 2.43 (2.24, 2.64) |
| None (reference) |  | 1.00 |  | 1.00 |
| **Season of landing date** |  |  |  |  |
| Autumn |  | 1.00 (0.98, 1.03) |  |  |
| Spring |  | 0.96 (0.94, 0.99) |  |  |
| Summer |  | 0.97 (0.94, 0.99) |  |  |
| Winter (reference) |  | 1.00 |  |  |
| **Time to travel to a CHC** |  |  |  |  |
| 3 minutes |  |  |  | 2.17 (1.96, 2.40) |
| 3 - 10 minutes |  |  |  | 2.69 (2.45, 2.96) |
| > 10 minutes (reference) |  |  |  | 1.00 |

^1^ Those living in rural areas were excluded due to low numbers.

^2^ Those with missing postal codes were excluded.

^3^ Includes those with suppressed deprivation data.

^4^ Includes missing data due to small numbers (cell sizes <6)
