# Supplementary material for: Primary health care utilization in the first year after arrival by refugee sponsorship model in Ontario, Canada: A population-based cohort study
Source: PLoS One. 2023 Jul 26;18(7):e0287437. doi: 10.1371/journal.pone.0287437 (PMC10370760; doi:10.1371/journal.pone.0287437)
Supplement: S8 Table — (DOCX) [file pone.0287437.s009.docx]

S8 Table: **Cox proportional hazards ratios for the association between sponsorship model and country cohort on time to first primary care (PC) and logistic odds ratios for the association between sponsorship model and country cohort on any community health centre (CHC) visit in the first year of resettlement in all resettled refugees who landed in Ontario between April 1, 2008 and March 31, 2017 including adjustment for landing year.**

|  | **Time to first PC visit (days)^1^ N=58,601** | | **Odds of a CHC visit in year 1^2^ N=58,477** | |
| --- | --- | --- | --- | --- |
| **Covariate** | **Unadjusted HR (95% CI)** | **Adjusted HR (95% CI)** | **Unadjusted OR (95% CI)** | **Adjusted OR (95% CI)** |
| **Country cohort + Sponsorship model** |  |  |  |  |
| Syria – GARs | 2.39 (2.28, 2.51) | 2.13 (2.02, 2.24) | 5.07 (4.32, 5.95) | 3.37 (2.83, 4.01) |
| Syria – PSRs | 1.39 (1.32, 1.46) | 1.22 (1.16, 1.29) | 1.53 (1.29, 1.82) | 1.17 (0.98, 1.41) |
| Afghanistan - GARS | 1.89 (1.76, 2.03) | 2.29 (2.12, 2.47) | 2.07 (1.64, 2.60) | 3.98 (3.12, 5.06) |
| Afghanistan - PSRS | 0.92 (0.87, 0.97) | 1.03 (0.97, 1.10) | 0.43 (0.33, 0.56) | 0.62 (0.48, 0.81) |
| Other African - GARs | 1.71 (1.58, 1.85) | 1.86 (1.72, 2.02) | 3.53 (2.83, 4.41) | 5.48 (4.35, 6.91) |
| Other African - PSRs | 1.13 (1.02, 1.25) | 1.27 (1.15, 1.40) | 0.73 (0.49, 1.09) | 0.95 (0.64, 1.41) |
| Bhutan - GARs³ | 1.33 (1.23, 1.43) | 1.57 (1.46, 1.70) | 5.49 (4.50, 6.69) | 10.01 (8.09, 12.4) |
| Congo - GARS | 1.77 (1.64, 1.90) | 2.05 (1.90, 2.20) | 7.32 (6.03, 8.88) | 10.35 (8.42, 12.73) |
| Congo - PSRS | 0.88 (0.76, 1.02) | 0.96 (0.83, 1.11) | 0.58 (0.31, 1.07) | 0.65 (0.35, 1.23) |
| Eritrea - GARs | 1.94 (1.73, 2.17) | 2.10 (1.87, 2.35) | 3.05 (2.25, 4.13) | 3.45 (2.52, 4.73) |
| Eritrea - PSRs | 0.86 (0.80, 0.91) | 0.87 (0.82, 0.93) | 1.10 (0.88, 1.38) | 0.99 (0.79, 1.25) |
| Ethiopia - GARs | 2.15 (1.93, 2.39) | 2.62 (2.35, 2.92) | 1.67 (1.18, 2.35) | 2.52 (1.77, 3.6) |
| Ethiopia - PSRs | 0.78 (0.71, 0.86) | 0.92 (0.83, 1.01) | 0.84 (0.60, 1.19) | 1.29 (0.91, 1.84) |
| Iran - GARS | 2.32 (2.17, 2.47) | 2.64 (2.47, 2.82) | 2.29 (1.87, 2.81) | 3.61 (2.92, 4.48) |
| Iran - PSRS | 1.24 (1.07, 1.44) | 1.41 (1.22, 1.64) | 1.42 (0.88, 2.28) | 2.09 (1.28, 3.42) |
| Iraq - GARs | 2.29 (2.18, 2.40) | 2.56 (2.44, 2.69) | 1.35 (1.13, 1.60) | 1.98 (1.66, 2.37) |
| Iraq - PSRs | 0.97 (0.93, 1.02) | 1.14 (1.08, 1.20) | 0.11 (0.09, 0.15) | 0.21 (0.16, 0.28) |
| Myanmar - GARs | 1.60 (1.48, 1.73) | 1.89 (1.75, 2.05) | 4.82 (3.93, 5.91) | 9.04 (7.25, 11.28) |
| Myanmar - PSRs | 0.75 (0.65, 0.87) | 0.84 (0.72, 0.97) | 2.73 (1.93, 3.86) | 3.6 (2.52, 5.15) |
| Somalia - GARS | 1.91 (1.79, 2.04) | 2.38 (2.22, 2.54) | 3.13 (2.58, 3.80) | 5.97 (4.86, 7.34) |
| Somalia - PSRS | 0.73 (0.67, 0.80) | 0.89 (0.81, 0.97) | 0.86 (0.64, 1.17) | 1.24 (0.91, 1.69) |
| Other GARs | 1.88 (1.76, 2.01) | 2.10 (1.96, 2.25) | 2.70 (2.20, 3.31) | 3.6 (2.91, 4.44) |
| Other PSRs (reference) | 1.00 | 1.00 | 1.00 | 1.00 |
| **Age group in years** |  |  |  |  |
| 0 to 5 |  | 1.25 (1.21, 1.29) |  | 0.93 (0.85, 1.02) |
| 6 to 11 |  | 1.02 (0.99, 1.05) |  | 0.91 (0.83, 0.99) |
| 12 to 17 |  | 0.93 (0.90, 0.96) |  | 0.88 (0.8, 0.97) |
| 18 to 30 (reference) |  | 1.00 |  |  |
| 31 to 45 |  | 1.22 (1.19, 1.26) |  | 1.14 (1.05, 1.24) |
| 46 to 65 |  | 1.42 (1.37, 1.46) |  | 1.15 (1.04, 1.28) |
| 66 to 100 |  | 1.63 (1.55, 1.73) |  | 1.15 (0.95, 1.4) |
| **Sex** |  |  |  |  |
| Female |  | 1.15 (1.13, 1.17) |  | 1.07 (1.01, 1.13) |
| Male (reference) |  | 1.00 |  | 1.00 |
| **Neighborhood Deprivation Quintile** |  |  |  |  |
| Q1 - least deprived |  | 1.05 (0.99, 1.12) |  | 0.88 (0.7, 1.11) |
| Q2 |  | 1.00 (0.95, 1.04) |  | 1.15 (0.98, 1.34) |
| Q3 |  | 1.02 (0.99, 1.06) |  | 1.01 (0.9, 1.13) |
| Q4 |  | 1.10 (1.07, 1.12) |  | 0.81 (0.76, 0.87) |
| Q5 - most deprived (reference)³ |  | 1.00 |  | 1.00 |
| **Canadian language ability** |  |  |  |  |
| English and/or French |  | 0.92 (0.90, 0.94) |  | 0.81 (0.76, 0.87) |
| None (Reference)⁴ |  | 1.00 |  | 1.00 |
| **Secondary immigration** |  |  |  |  |
| Yes |  | 1.04 (1.01, 1.07) |  | 1.22 (1.1, 1.34) |
| None (reference) |  | 1.00 |  | 1.00 |
| **Season of landing date** |  |  |  |  |
| Autumn |  | 1.00 (0.98, 1.03) |  | 1.19 (1.09, 1.29) |
| Spring |  | 0.96 (0.94, 0.99) |  | 1.14 (1.06, 1.23) |
| Summer |  | 0.97 (0.94, 0.99) |  | 0.97 (0.89, 1.06) |
| Winter (reference) |  | 1.00 |  | 1.00 |
| **Landing Year** |  |  |  |  |
| 2008 |  | 0.71 (0.67, 0.74) |  | 0.58 (0.5, 0.67) |
| 2009 |  | 0.72 (0.68, 0.75) |  | 0.2 (0.17, 0.24) |
| 2010 |  | 0.66 (0.63, 0.70) |  | 0 (0, 0) |
| 2011 |  | 0.68 (0.65, 0.71) |  | 0.21 (0.17, 0.25) |
| 2012 |  | 0.67 (0.64, 0.70) |  | 0.35 (0.30, 0.41) |
| 2013 |  | 0.75 (0.72, 0.78) |  | 0.55 (0.48, 0.62) |
| 2014 |  | 0.91 (0.88, 0.95) |  | 0.66 (0.59, 0.74) |
| 2015 |  | 0.94 (0.91, 0.96) |  | 0.95 (0.89, 1.02) |
| 2016 (reference) |  | 1.00 |  | 1.00 |
| **Time to travel to a CHC** |  |  |  |  |
| 3 minutes |  |  |  | 2.21 (2.00, 2.45) |
| 3 - 10 minutes |  |  |  | 2.67 (2.43, 2.94) |
| > 10 minutes (reference) |  |  |  | 1.00 |

1 Those living in rural areas were excluded due to low numbers.

2 Those with missing postal codes were excluded.

3 Includes those with suppressed deprivation data.

4 Includes missing data due to small numbers (cell sizes <6)
