# Supplementary material for: Primary health care utilization in the first year after arrival by refugee sponsorship model in Ontario, Canada: A population-based cohort study
Source: PLoS One. 2023 Jul 26;18(7):e0287437. doi: 10.1371/journal.pone.0287437 (PMC10370760; doi:10.1371/journal.pone.0287437)
Supplement: S9 Table — (DOCX) [file pone.0287437.s010.docx]

S9 Table. **Cox proportional hazards estimates of the association between sponsorship model and era on primary care (PC) and logistic odds ratio for any community health centre (CHC) visit in the first year of resettlement in all resettled refugees who landed in Ontario between April 1, 2008 and March 31, 2017 among those aged ≥ 25.**

|  | **Time to first PC visit (days)^1^ N=58,601** | | **Odds of a CHC visit in year 1^2^ N=58,477** | |
| --- | --- | --- | --- | --- |
| **Covariate** | **Unadjusted HR (95% CI)** | **Adjusted HR (95% CI)** | **Unadjusted OR (95% CI)** | **Adjusted OR (95% CI)** |
| **Era of landing + Sponsorship model** |  |  |  |  |
| Syrian Era - Syrian GARs | 3.08 (2.95, 3.21) | 2.55 (2.42, 2.69) | 17.78 (15.26, 20.71) | 17.33 (14.44, 20.81) |
| Syrian Era - Syrian BVORs | 2.41 (2.22, 2.61) | 1.99 (1.83, 2.17) | 15.46 (12.49, 19.14) | 17.15 (13.54, 21.74) |
| Syrian Era - Syrian PSRs | 1.35 (1.30, 1.41) | 1.10 (1.05, 1.16) | 2.40 (1.97, 2.92) | 2.62 (2.11, 3.25) |
| Syrian Era - Non-Syrian GARs | 2.91 (2.72, 3.10) | 2.77 (2.58, 2.96) | 18.36 (15.24, 22.12) | 11.38 (9.31, 13.91) |
| Syrian Era - Non-Syrian BVORs | 2.13 (1.82, 2.49) | 1.97 (1.68, 2.31) | 13.68 (9.51, 19.69) | 7.65 (5.22, 11.22) |
| Syrian Era - Non-Syrian PSRs | 1.19 (1.13, 1.26) | 1.19 (1.12, 1.26) | 2.09 (1.63, 2.67) | 0.95 (0.74, 1.23) |
| Pre-Syrian Era GARs | 2.40 (2.32, 2.48) | 2.34 (2.27, 2.42) | 6.77 (5.84, 7.85) | 6.57 (5.63, 7.66) |
| Pre-Syrian Era PSRs (reference) | 1.00 | 1.00 | 1.00 | 1.00 |
| **Age group in years** |  |  |  |  |
| 25 to 35 (reference) |  | 1.00 |  | 1.00 |
| 36 to 45 |  | 1.06 (1.02, 1.09) |  | 1.06 (1.02, 1.09) |
| 46 to 65 |  | 1.22 (1.18, 1.26) |  | 1.22 (1.18, 1.26) |
| 66 to 100 |  | 1.43 (1.35, 1.52) |  | 1.43 (1.35, 1.52) |
| **Sex** |  |  |  |  |
| Female |  | 1.26 (1.23, 1.29) |  | 1.13 (1.04, 1.22) |
| Male (reference) |  | 1.00 |  | 1.00 |
| **Neighborhood Deprivation Quintile** |  |  |  |  |
| Q1 - least deprived |  | 1.06 (0.98, 1.15) |  | 1.13 (0.83, 1.53) |
| Q2 |  | 0.99 (0.93, 1.06) |  | 1.27 (1.01, 1.6) |
| Q3 |  | 1.03 (0.98, 1.08) |  | 1.14 (0.96, 1.34) |
| Q4 |  | 1.08 (1.05, 1.11) |  | 0.76 (0.69, 0.84) |
| Q5 - most deprived (reference)³ |  | 1.00 |  | 1.00 |
| **Canadian language ability** |  |  |  |  |
| English and/or French |  | 0.97 (0.94, 1.00) |  | 0.93 (0.85, 1.02) |
| None (Reference)⁴ |  | 1.00 |  | 1.00 |
| **World Region of Citizenship** |  |  |  |  |
| Africa |  | 1.03 (0.98, 1.08) |  | 0.92 (0.8, 1.06) |
| Americas |  | 1.17 (1.00, 1.36) |  | 1.61 (1.12, 2.32) |
| Asia & Pacific (reference) |  | 1.00 |  | 1.00 |
| Europe & USA |  | 0.80 (0.63, 1.00) |  | 0.35 (0.17, 0.76) |
| Middle East |  | 1.16 (1.12, 1.21) |  | 0.33 (0.28, 0.37) |
| Stateless⁴ |  | 0.91 (0.8, 1.04) |  | 0.96 (0.68, 1.37) |
| **Secondary migration** |  |  |  |  |
| Yes |  | 1.19 (1.15, 1.23) |  | 2.74 (2.43, 3.09) |
| None (reference) |  | 1.00 |  | 1.00 |
| **Season of landing date** |  |  |  |  |
| Autumn |  | 1.02 (0.98, 1.05) |  |  |
| Spring |  | 1.00 (0.97, 1.04) |  |  |
| Summer |  | 0.98 (0.95, 1.02) |  |  |
| Winter (reference) |  | 1.00 |  |  |
| **Time to travel to a CHC** |  |  |  |  |
| 3 minutes |  |  |  | 2.85 (2.45, 3.31) |
| 3 - 10 minutes |  |  |  | 2.98 (2.58, 3.44) |
| > 10 minutes (reference) |  |  |  | 1.00 |
| **Marital Status** |  |  |  |  |
| Single |  | 0.76 (0.73, 0.78) |  | 0.91 (0.8, 1.02) |
| Separated/widowed/divorced⁴ |  | 0.91 (0.86, 0.95) |  | 0.90 (0.77, 1.04) |
| Married (reference) |  | 1.00 |  | 1.00 |
| **Highest education level** |  |  |  |  |
| Secondary or less⁴ |  | 0.91 (0.88, 0.95) |  | 0.86 (0.75, 0.99) |
| Trade or Diploma, or some University (less than Bachelor’s) |  | 0.98 (0.93, 1.03) |  | 0.85 (0.71, 1.02) |
| Bachelor's of higher (reference) |  | 1.00 |  | 1.00 |
