# Supplementary material for: Primary health care utilization in the first year after arrival by refugee sponsorship model in Ontario, Canada: A population-based cohort study
Source: PLoS One. 2023 Jul 26;18(7):e0287437. doi: 10.1371/journal.pone.0287437 (PMC10370760; doi:10.1371/journal.pone.0287437)
Supplement: Attachment — Submitted filename: 20230419_PLOSONE_response_tables2.docx [file pone.0287437.s011.docx]

PLOSONE Response to Reviewers – ***PLEASE NOTE* that all line numbers indicated refer to the document with no tracked changes**

Journal Requirements

| # | Reviewer Comment | Author Response |
| --- | --- | --- |
| 1 | Please ensure that your manuscript meets PLOS ONE's style requirements, including those for file naming. | This has been completed. |
| 2 | 2. Please provide additional details regarding participant consent. In the ethics statement in the Methods and online submission information, please ensure that you have specified (1) whether consent was informed and (2) what type you obtained (for instance, written or verbal, and if verbal, how it was documented and witnessed). If your study included minors, state whether you obtained consent from parents or guardians. If the need for consent was waived by the ethics committee, please include this information.  If you are reporting a retrospective study of medical records or archived samples, please ensure that you have discussed whether all data were fully anonymized before you accessed them and/or whether the IRB or ethics committee waived the requirement for informed consent. If patients provided informed written consent to have data from their medical records used in research, please include this information. | The following language has now been included on the online submission system - “ICES is a prescribed entity under Ontario’s Personal Health Information Protection Act (PHIPA). Section 45 of PHIPA authorizes ICES to collect personal health information, without consent, for the purpose of analysis or compiling statistical information with respect to the management of, evaluation or monitoring of, the allocation of resources to or planning for all or part of the health system. Projects that use data collected by ICES under section 45 of PHIPA, and use no other data, are exempt from REB review. The use of the data in this project is authorized under section 45 and approved by ICES’ Privacy and Legal Office. The data were fully anonymized and used an encrypted unique identifier to link across databases prior to analysis.”  The “Ethics approval” sub-section of the Methods in the manuscript (lines 276-277) now includes the following sentence – “The data were fully anonymized and used an encrypted unique identifier to link across databases prior to analysis.” |
| 3 | 3. PLOS requires an ORCID iD for the corresponding author in Editorial Manager on papers submitted after December 6th, 2016. Please ensure that you have an ORCID iD and that it is validated in Editorial Manager. To do this, go to ‘Update my Information’ (in the upper left-hand corner of the main menu), and click on the Fetch/Validate link next to the ORCID field. This will take you to the ORCID site and allow you to create a new iD or authenticate a pre-existing iD in Editorial Manager. Please see the following video for instructions on linking an ORCID iD to your Editorial Manager account: [https://www.youtube.com/watch?v=_xcclfuvtxQ"](https://can01.safelinks.protection.outlook.com/?url=https%3A%2F%2Furldefense.com%2Fv3%2F__https%3A%2Fwww.youtube.com%2Fwatch%3Fv%3D_xcclfuvtxQ__%3B!!D0zGoin7BXfl!5Jgcy0ztCIq9iNVhzrv9H4PmsK14ecotEcq9tMhvng1hJ7V1cyzqKyY4KFd72QMHCEDPixFqxccdKMH8WWfVAg2Bi7Q%24&data=05%7C01%7Castrid.guttmann%40ices.on.ca%7C7e0e4bfefa044e64455e08db3126db03%7C96cfb6930adb463ba787339492037306%7C0%7C0%7C638157815664258781%7CUnknown%7CTWFpbGZsb3d8eyJWIjoiMC4wLjAwMDAiLCJQIjoiV2luMzIiLCJBTiI6Ik1haWwiLCJXVCI6Mn0%3D%7C3000%7C%7C%7C&sdata=Uzi5ZPRGoRGUYMXOkwmxqRWnpIuJBpD80Iq%2FHp8cx%2BU%3D&reserved=0) | The corresponding author’s ORCID ID has been added. |
| 4 | 4. Please update your submission to use the PLOS LaTeX template. The template and more information on our requirements for LaTeX submissions can be found at [http://journals.plos.org/plosone/s/latex.](https://can01.safelinks.protection.outlook.com/?url=https%3A%2F%2Furldefense.com%2Fv3%2F__http%3A%2Fjournals.plos.org%2Fplosone%2Fs%2Flatex.__%3B!!D0zGoin7BXfl!5Jgcy0ztCIq9iNVhzrv9H4PmsK14ecotEcq9tMhvng1hJ7V1cyzqKyY4KFd72QMHCEDPixFqxccdKMH8WWfVD8tTGRY%24&data=05%7C01%7Castrid.guttmann%40ices.on.ca%7C7e0e4bfefa044e64455e08db3126db03%7C96cfb6930adb463ba787339492037306%7C0%7C0%7C638157815664258781%7CUnknown%7CTWFpbGZsb3d8eyJWIjoiMC4wLjAwMDAiLCJQIjoiV2luMzIiLCJBTiI6Ik1haWwiLCJXVCI6Mn0%3D%7C3000%7C%7C%7C&sdata=Ab64vdaLhV5I87K%2B1%2BGlY86FjngXHMVXKtWsm3uF5bY%3D&reserved=0) | Our original submission was in PDF for the ease of the review process but we will be submitting the final documents in a Microsoft Word Document format. |
| 5 | 5. Please review your reference list to ensure that it is complete and correct. If you have cited papers that have been retracted, please include the rationale for doing so in the manuscript text, or remove these references and replace them with relevant current references. Any changes to the reference list should be mentioned in the rebuttal letter that accompanies your revised manuscript. If you need to cite a retracted article, indicate the article’s retracted status in the References list and also include a citation and full reference for the retraction notice. | The reference list has been reviewed to ensure it is complete and correct. |

Reviewer #1:

Thank you for the invitation to review this work. I have accepted this invitation mainly out of curiosity, not being an expert in the field, but being an involved world citizen, and general practitioner working in the Netherlands, where I encounter different problems with the access to care of refugees. So this is also my disclaimer. I do, however, also have methodological skills as an epidemiologist with which I give my feedback to this manuscript. I have not encountered methodological major issues, but only minor comments.

| # | Reviewer Comment | Author Response |
| --- | --- | --- |
| 1 | In general, the manuscript is very long due to the details shared. The advantage of this, is the transparency, but the disadvantage is the difficulty to remain focussed while reading. | I have gone through the manuscript in detail and have cut down on extraneous words throughout. Some details on these changes are described here. Supplementary Table 1 is now referred to at the beginning of the methods/data sources section so now only the immigration database and healthcare registry are described here. In the methods section, in describing the variables (exposures, outcomes, covariates) the text has been substantially reduced with the removed details now included in a new Supplementary Table 2. The results section has also been slightly condensed as has the discussion. Current word count is ~4400 words. |
| 2 | Next, I feel that the conclusions drawn (as described in the abstract) go beyond the scope of this article. Specifically, as it is unknown what the content of the consultations with the CHC were. | This sentence has been removed from the abstract and replaced with the following (line 44) – “Less timely primary care and lower odds of a CHC visit among PSRs in the first year may be attributed to selection factors and gaps in sponsors’ knowledge of healthcare navigation.” This is consistent with changes made to a similar statement in the discussion and conclusion in the body of the manuscript, as per comment #10. |
| 3 | Although abbreviations are explained, the use of many abbreviations (PSR BVOR GARS) is a bit confusing for an outsider. | Thank you for this comment. Since these terms are very specific to Canada, aside from the abstract and tables and figures, we have removed use of these acronyms and have expanded them to use the full terms. |
| 4 | At the end of the introduction, two hypothesis are mentioned. The first is clear and explained, but the second remains unclear to me, as I don’t truly see why the authors mean with “.., related to the higher profile government, civic and professional response to resettling those fleeing the Syrian conflict” (line 137-8) Please elaborate a bit more to keep the reader focussed. | The reason for the second hypothesis is elaborated on in the first paragraph of the introduction (lines 54-57) which now reads “In response to the Syrian migrant crisis, starting in late 2015 Canada accepted over 40,000 refugees within an 18-month period. The Syrian response was characterized by unprecedented mobilization and engagement of provincial governments (including their health systems), the settlement sector and civil society.”  We have also referenced recently released reports that describe the substantial govt and civic response (Syrian Lived Experience Project, 2022 + Syrian outcomes report, 2019). The last paragraph of the introduction (lines 121-124) which describes the second hypothesis now reads – “Our second hypothesis is that refugees resettled during the Syrian aera, particularly those from Syria, compared to those resettled in the pre-Syrian era, will have more timely primary care access related to the increased civic and government engagement in response to the Syrian migrant crisis.” |
| 5 | In the methods section, authors provide detailed information on the data sources, and define “linked and unlinked refugees” meaning (if my interpretation is correct) that refugees were not included in the RPDB (unlinked refugees). It is unclear WHEN people are registered in that database. Is a direct contact with a HCP obligatory for that? So, are the unlinked refugees people who haven’t had a single contact in the study period? | People are registered for the healthcare registry (RPDB) when they become eligible for healthcare and have received a health card number. Registration is not dependent on contact with a healthcare provider.  Unlinked immigrants and refugees are those who i) have identifying information in the immigration database which does not match to any record in the healthcare registry or ii) did not become a resident of Ontario (e.g., may have moved to another Canadian province) and therefore did not receive a health card number. New health care registry data are received each year; and each year these data are linked to the immigration database at ICES to update the linked data repository (this is not just done for specific studies).  In S1 Fig we report the overall (deterministic + probabilistic) linkage rate of the immigration database (1985-2017) to the healthcare registry used in our study, which was 86.8%. We reference a peer-reviewed study (Chiu et al, 2016) which describes the linkage methodology in detail. The linkage statistics in S1 Fig are similar but not identical to those reported in Chiu et al, 2016 because the linked datasets used in our study include additional years of data that were not available at the time of the published study; however the linkage methodology described in the published study is the same that was used in our study  To clarify the above, on line 141-150, I have added more details which now reads – “Resettled refugees were identified from the ICES data repository which links the Ontario portion of the Immigration, Refugees and Citizenship Canada (IRCC) Permanent Resident Database to the healthcare registry. About 92% of refugees (resettled refugees and protected persons) are linked and included in the ICES data repository (see S1 Fig for linkage information). The healthcare registry includes demographic information and vital statistics on all Ontario residents who have been eligible for publicly funded healthcare and have received a health card number since 1990. This single-payer system funds access to most medically necessary health-care services. There are few differences in characteristics between refugees linked to the healthcare registry compared to those unlinked [26], suggesting minimal linkage bias. The study cohort was then deterministically linked to healthcare databases using unique encoded identifiers.” |
| 6 | It could be helpful to provide a Figure clarifying how data bases were combined. It seems that Figure 1 in the supplementary files have less databases mentioned than the method section. | Appendix Figure 1 has been updated to clarify how all databases were combined and explanatory text has been included (as described above on lines 141-150). |
| 7 | Please acknowledge that due to the very large number of patients included in the database, the change of having a statistically significant difference for any comparison is large. | On line 189-190 now includes the following sentence - “Given the large population sizes we acknowledge that small differences in the outcomes will be significantly different therefore our interpretation is informed by clinical, rather than statistical, significance.” |
| 8 | The clinical relevance of difference needs more attention. | See answer to Q 7 above. We have also removed p-values from Table 3 to signal reduced importance of p-values and use a subscript to signify statistical significance in its place. The description of table 3 (lines 237-255) in the results section describes clinically relevant results. |
| 9 | Here, authors have chosen to present Odds ratios (line 324-332) where relative risk ratios would be more appropriate, taken into consideration the very high prevalence of a CHC visit in this population. | We agree with the reviewer. In the methods section on lines 203-205 we now include the following caveat – “We recognized that if CHC visits are prevalent, logistic regression models will overestimate relative risks therefore we report odds rather than risks or probability.” |
| 10 | In the discussion, at some points I don’t follow the argumentation of the authors. For example, in line 369-370, authors mention “While morbidity was lower among PSRs, lower primary care use in the first year of resettlement may have important implications for prevention, quality of primary care and longer-term adverse health outcomes.” As a GP I disagree with that statement. Why would GPs start preventative measures when a patient visits the practice, e.g. for headache or a skin problem. This suggestions goes beyond the scope of this study, and I would suggest the authors to refrain from expressing this kind of unsupported thoughts. | This sentence in the discussion has been removed.  In addition, in the conclusion (line 460-461) the bolded part of the sentence following has been removed. “…, may signal gaps in sponsors’ knowledge of healthcare **needs and** service[s] **access** **with additional implications for prevention and development of longer term adverse health outcomes**.”  I have also edited throughout the discussion in attempts to make clearer arguments. |

Reviewer #2:

| # | Reviewer Comment | Author Response |
| --- | --- | --- |
| 1 | - Associations "between" not "of", as the latter implies that the object of study is a formal administrative structure between the two, whilst the former implies a statistical association. - Replace numerical "1st" with "first" - Maybe a reformulation would be more clear. Something along the lines of "Primary health care utilization amongst refugees in their first year in Canada: a population-based cohort study" | The title of the manuscript has been changed to “Primary health care utilization in the first year after arrival by refugee sponsorship model in Ontario, Canada: a population-based cohort study”  1^st^ has also been changed to “first” throughout the manuscript |
| 2 | Keywords - some of the selected keywords are a bit unusual and/or non-specific, e.g., "centres, community health". The authors are encouraged to reconsider these carefully. | The keywords entered initially are all MESH terms but alternatives have been chosen. As per the reviewer’s suggestion instead of “centers, community health”, “community health center” has been chosen. “Ontario” has also been removed as a key word but “Canada” retained. |
| 3 | - In-text citation is inconsistent (e.g., ln 66 & ln 68). Please double-check for formatting, also in the references list.  - There are a few writing errors scattered throughout, e.g., ln 87 comma splice. The authors should conduct a thorough final revision for writing. | All the references have been double checked for formatting in the manuscript. |
| 4 | - The writing is, at times, unclear. The authors are strongly advised to consider revising the manuscript for clarity. | We have done an additional edit for clarity – both cutting words and reorganizing parts of the manuscript. We have reduced words and re-organized the introduction to improve flow. Parts of the methods section have been moved to a supplementary table (supplementary table 2). The discussion has also been edited to improve clarity. |
| 5 | - Is the Canadian mixed sponsorship model entirely unique? I was under the impression that a mixed model is also used in some European countries, but I might be wrong. If there are any other models that are somewhat similar, e.g., following the influx of refugees fleeing from Ukraine into the EU/EEA, these should be differentiated in the first paragraph of the background section. | Canada pioneered the private sponsorship program (which began in 1979) and the Global Sponsorship Initiative has been working to encourage private sponsorship programs in other countries, specifically the approach where refugees are chosen by the UNHCR or a migration agency and private sponsoring groups are NOT friends or family members of the refugees being sponsored (the arrangement for financial support is flexible meaning it can be both govt and sponsoring group or just the sponsoring group). The approach being promoted internationally is referred to as “community-sponsored” and has now been implemented in several countries. In response to the Ukraine war the United States has also launched a private sponsorship program in January 2023.  These details have been edited/added to the first paragraph of the background section (line 60) which now reads “Given the global demand for resettlement, the Global Refugee Sponsorship initiative [9] has helped spread Canada’s private sponsorship program, specifically the approach where refugees are referred by the UNHCR or migration agency and private sponsors are unknown to the refugees they sponsor. Such programs have been implemented in several other countries including the United Kingdom, New Zealand and most recently to the United States [10,11].  In addition, the word “unique” has also been removed from the title of the manuscript to reflect that many other countries now have private sponsorship programs for refugees. |
| 6 | - Experimental setup is very well designed and executed, including selection of criteria/variables. - Correct selection and use of statistical tests. - Results are presented clearly and logically and the tables provide useful summaries of the results. - Interpretation of results is clear and does not meander into unqualified speculation. - Study limitations appropriately reflected. | Thank you for these comments, much appreciated. |
| 7 | - Table 1 is very useful. The authors should refer to it earlier in the text. The footnotes for Table 1 should be separated by line breaks. | Line breaks now included. |
| 8 | - Background section very informative! | Great! |
| 9 | - Background ln 128, indicate what is defined as the pre-Syrian period. April 1, 2008 to Nov 2015? | Yes, the reviewer is correct. This has been added on line 113. |
| 10 | - Paragraph starting on ln 144: indicate the number of excluded cases for each exclusion criterion! | These numbers have now been included in the first paragraph of the methods section (line 129) along with a reference to (an updated) Appendix figure 1 which had these numbers included. This reads – “We excluded those without a valid Ontario health card number (N=329, 0.55%) and a small number of records for individuals who had more than one arrival record with the arrival before April 1, 2008 (N=24, 0.04%) to limit our sample to those new to the Ontario healthcare system. See S1 Figure for database linkage and cohort selection flowchart.” |
| 11 | - Para. Starting on ln 171: please define “era of landing” as a binary variable and explain in the following sentence that it pertains to the war in Syria. The reasoning for the exact dates will need to be provided. | In the new Supplementary table 2, “Era of landing” is described as binary.  Additional text describing the reasoning for these periods reads (lines 155-158) – “ The pre-Syrian era’s large resettled refugee population served as a comparison for those landing in the Syrian-era which marked the start of the Government of Canada’s commitment to resettle Syrian refugees who had fled to neighbouring countries.” |
| 12 | - ln 207, are adults defined as >18 or >=18 years of age? In most cases, 18 year-olds are considered legal adults in Canada, to my knowledge. | This was an error and should have read >=18 years, now corrected. Thank you! |
| 13 | - Did the authors use census neighbourhoods (do these exist?) or census dissemination areas (DAs)? They look to me like DAs. | The marginalization index is measured at the census dissemination area-level and are not census “neighborhoods” by which a person may define the community in which they reside.  The description of material deprivation now included in supplementary table 2 reflects that the areas for which it is measured are not neighborhoods but census areas. The text in the manuscript (line 176) now reads “…census are-level material deprivation [34].” |
| 14 | - Replace instances of 1st with first in text, e.g., ln 296. | Thank you – this has been done throughout*.* |
| 15 | _ I wonder to what degree in-community health care (i.e., without interfacing with the public health care system in Ontario) plays a role and how this might differ between various subpopulations? | In Ontario there is no “in-community health care” as described by the comment. The province of Ontario’s healthcare system is a single-payer (i.e., the Government of Ontario, Ministry of Health), publicly funded (through taxation) system. The vast majority of primary and acute healthcare delivered to Ontarians is through this publicly funded system, including healthcare services offered through Community Health Centres. In case the reviewer is thinking of Community Health Centres as referred to in our study, as “in-community health care”, we’ll take this opportunity to describe them in more detail here. Community Health Centres offer an interdisciplinary model of primary care which includes physicians and other allied healthcare professionals. Unique to primary care settings, some community health centers also have settlement services and other community-based services which are funded through mechanism that differ than those that fund healthcare services (e.g., federal settlement funding).  We have edited the paragraph in the introduction which describes primary care in Ontario to make this clearer (lines 99-109). This now reads – “In Ontario, all resettled refugees are eligible for publicly funded provincial healthcare insurance on arrival. For recent refugees who often have unmet healthcare needs, primary care is particularly important. Primary care is central to screening, prevention and treatment and is the gateway to specialized medical care. In Ontario, there are distinct models of primary care delivery with about 75% of Ontario’s population belonging to a Patient Enrollment Model (PEM), often associated with comprehensive primary care [20,21]. Community Health Centres (CHCs) include a specialized model of primary care integrated with social services and community programming [22] serving Ontarians facing barriers. Several CHCs are specifically oriented to immigrant health, including formal settlement services in-house and access to interpretation services making it easier for immigrants to connect to and navigate primary health care services.” |
